# Multiple Tick-Borne Pathogens in *Ixodes ricinus* Ticks Collected from Humans in Romania

**DOI:** 10.3390/pathogens9050390

**Published:** 2020-05-19

**Authors:** Zsuzsa Kalmár, Mirabela Oana Dumitrache, Gianluca D’Amico, Ioana Adriana Matei, Angela Monica Ionică, Călin Mircea Gherman, Mihaela Lupșe, Andrei Daniel Mihalca

**Affiliations:** 1 Department of Parasitology and Parasitic Diseases, Faculty of Veterinary Medicine, University of Agricultural Sciences and Veterinary Medicine Cluj-Napoca, 400372 Cluj-Napoca, Romania; mirabela.dumitrache@usamvcluj.ro (M.O.D.); gianluca.damico@usamvcluj.ro (G.D.); matei.ioana@usamvcluj.ro (I.A.M.); ionica.angela@usamvcluj.ro (A.M.I.); calin.gherman@usamvcluj.ro (C.M.G.); amihalca@usamvcluj.ro (A.D.M.); 2 Department of Microbiology, Immunology and Epidemiology, Faculty of Veterinary Medicine, Calea Mănăştur, no. 3–5, 400372 Cluj-Napoca, Romania; 3 “Regele Mihai I al României” Life Sciences Institute, University of Agricultural Sciences and Veterinary Medicine Cluj-Napoca, 3–5 Calea Mănăștur, 400372 Cluj-Napoca, Romania; 4 Department of Infectious Diseases, University of Medicine and Pharmacy “Iuliu Haţieganu”, Iuliu Moldovan 23, 400000 Cluj-Napoca, Romania; mihaela.lupse@yahoo.com

**Keywords:** *Ixodes ricinus*, ticks, pathogens, tick-borne disease, humans

## Abstract

Ticks are medically important vectors of infectious diseases that are able to transmit pathogens to humans and animals. Tick-borne diseases represent a major health concern, posing an increasing risk to the public health during the last century and affecting millions of people. The aim of the current study was to provide epidemiological data regarding the presence of certain tick-borne pathogens in ticks feeding on humans in Romania. Overall, 522 *Ixodes ricinus* ticks collected from humans were screened for six pathogens: *Borrelia* spp., *Neoehrlichia mikurensis*, *Babesia* spp., *Coxiella* spp., *Bartonella* spp., and *Francisella tularensis*. Ticks attached to humans were collected between 2013–2015 in Cluj County, Romania. Conventional, nested and quantitative PCR were used to detect specific genetic sequences of each pathogen. For identifying the infectious agents, positive samples were sequenced. The infection prevalence was 21.07% from which 8.18% were mixed infections. The detected agents were *Borrelia* spp., *N. mikurensis* and *Babesia* spp. The present data reveal the endemic occurrence of potentially zoonotic pathogens in Romania. Revealing the current distribution of tick-borne pathogens in ticks collected from humans may provide new insights in understanding the complex ecology of tick-borne diseases and enlightens current knowledge about the infection prevalence at local, regional and national levels.

## 1. Introduction

Ticks of the Ixodidae family have medical and veterinary importance worldwide, mainly due to the transmission of tick-borne pathogens [1]. Pathogens are maintained in nature by ticks and vertebrates which may act as vectors and reservoirs of infectious agents. Important pathogens vectored by ticks, besides bacteria, are viruses and protozoa. Among the main bacterial pathogens transmitted by ticks are the causative agents of Lyme borreliosis, *Borrelia burgdorferi* sensu lato, and *B. miyamotoi*, which causes relapsing fever. Although infections with other tick-borne pathogens transmitted by *Ixodes ricinus*, such as *Neoehrlichia mikurensis*, several *Babesia* spp., *Bartonella* spp., *Francisella tularensis*, and *Coxiella burnetii* may cause human disease as well, they have been reported considerably less frequently.

The risk of acquiring infections with tick-borne pathogens is determined by the abundance of ticks in the specific geographical location, the density of reservoir hosts and the prevalence of infection in ticks and hosts [2]. Monitoring tick-borne pathogens in certain geographical areas is of particular importance to assess the fluctuation of human infection risks. Cluj County is situated in the north-western part of Romania, and due to the presence of hilly and mountainous relief it has a heterogeneous vegetation and a relatively high diversity of hosts for *I. ricinus* [3]. So far, studies conducted in Romania have evaluated the prevalence of *Borrelia* spp., *N. mikurensis*, *Anaplasma* spp., and *Rickettsia* spp. in *I. ricinus* [4,5,6,7,8]. The only study with a large dataset elaborated in Romania has evaluated the infection prevalence of *B. burgdorferi* s.l. in ticks collected from humans and in patients bitten by *Borrelia*-positive ticks [4,5].

Although there is a continuous increase in the prevalence of tick-borne diseases in Europe, few data on ticks and tick-related diseases in humans are available, while in Romania the information is even more limited. Therefore, the aim of the present study is to address the knowledge gap regarding the infection level of six different tick-borne pathogens in *I. ricinus* collected from human hosts. DNA samples of *I. ricinus* collected from humans from our previous study focused on *A. phagocytophilum* and *Rickettsia* spp. [7] were reused to assess the presence of the following tick-borne pathogens: *Borrelia* spp., *N. mikurensis*, *Babesia* spp., *Bartonella* spp., *Coxiella* spp. and *Francisella* spp.

## 2. Results

According to the morphological keys [9], a total of 522 ticks collected from humans were identified as *I. ricinus*. Only *I. ricinus* species were screened, as other tick species were very scarcely represented. Nymphs were the most common developmental stages found on humans (417/520, 79.89%, 95% CI: 76.23–83.10), followed by females (84/520, 16.09%; 95% CI: 13.19–19.49) and larvae (21/520, 4.02%; 95% CI: 2.65–6.07). No adult males were identified. Data regarding the overall infection prevalence of all the detected tick-borne pathogens (including *A. phagocytophilum* and *Rickettsia* spp.) in *I. ricinus* are detailed in Supplementary Materials 1 and 2. Here, we present the results of the current study. All the ticks were negative for *Bartonella* spp., *Coxiella* spp. and *Francisella* spp. In total, 110 (21.07%) out of 522 ticks were infected with at least one pathogen (Table 1), from which 8.18% were mixed infections (Table 2). Only mixed infections with pathogens from different genera were detected. Of the collected ticks, 23.82% of females, 20.86% of nymphs and 9.52% of the larvae were infected by at least one pathogen. Significant annual differences in infection prevalence (χ^2^ = 13.041, df = 2, *P* = 0.0015) were observed for nymphs with higher infection rate in the second study year.

The most prevalent pathogen detected was *Borrelia* spp. with a prevalence of 14.18% (74/522; 95% CI: 11.45–17.43). DNA of *Borrelia* spp. was detected in all developmental stages: larvae (2/21, 9.52%; 95% CI: 1.17–30.38), nymphs (56/417, 13.49%; 95% CI: 10.54–17.12) and females (16/84, 19.05%; 95% CI: 11.30–29.08) without statistical differences between stages (χ^2^ = 2.2032, df = 2, *P* = 0.3323). A percentage of 89.19% (66/74; 95% CI: 79.80–95.22) of the sequenced *Borrelia*-positive samples showed similarity with species of the *B. burgdorferi* s.l. complex and 10.81% (8/74; 95% CI: 0.78–3.00) with *B. miyamotoi* relapsing-fever spirochetes. From the *B. burgdorferi* s.l. complex, four species were identified, with *B. afzelii* the most frequently detected (29/66, 43.94%; 95% CI: 31.74–56.7). Fifteen *Borrelia*-positive samples were identified as *B. garinii* (22.73%; 95% CI: 13.31–34.7), followed by *B. lusitaniae* (13, 19.7%; 95% CI: 10.93–31.32) and *B. valaisiana* (9, 13.64%; 95% CI: 6.43–24.31). Among the 74 positive *Borrelia* spp. samples, seven (9.46%; 95%CI: 3.89–18.52) were mixed infections with pathogens of different genera. No significant increase in the prevalence of *Borrelia* spp. was observed over the three years (χ^2^ = 5.1185, df = 2, *P* = 0.0774).

The second most frequently identified pathogen was *N. mikurensis* with a prevalence of 5.94% (31/522; 95% CI: 4.21–8.31). Higher prevalence was detected in nymphs, followed by females (Table 1) (χ^2^ = 2.618, df = 2, *P* = 0.2701). None of the larvae were infected with this pathogen. A significant increase (χ^2^ = 11.2949, df = 2, *P* = 0.0035) in the prevalence of *N. mikurensis* was observed during the collection years, varying from 0.65% to 8.85%, as well in nymphs (χ^2^ = 11.8561, df = 2, *P* = 0.0027) with infection prevalence ranging between 0.72–9.94%.

DNA of *Babesia* spp. was detected in 2.87% of the ticks (15/522; 95% CI: 1.75–4.69). Infection with this genus was identified in each year. None of the larvae were *Babesia*-positive. Two *Babesia* species were identified, *B. microti* (2.11%; 95% CI: 1.18–3.73) and *B. venatorum* (0.77%; 95% CI: 0.30–1.95) (Table 1). Among these, 14 samples were positive in nymphs (3.36%; 95% CI: 2.01–5.56) and one female (1.19%; 95% CI: 0.03–6.46) (Table 1).

Mixed infections were detected only in nymphs, with 9 ticks out of the 110 infected (Table 2) harboring co-infections with various pathogens (9/417, 2.16%; 95% CI: 1.14–4.05). The following species combinations were detected: *B. afzelii*/*B. microti*, *B. afzelii*/*N. mikurensis*, *B. lusitaniae*/*N. mikurensis*, *B. valaisiana*/*N. mikurensis*, *N. mikurensis*/*B. microti*, *N. mikurensis*/*B. venatorum*. A co-infection with *B. valaisiana*/*N. mikurensis*/*B. venatorum* was detected in one nymph (Table 2).

## 3. Discussion

Studies on the presence and distribution of some tick-borne pathogens in humans and ticks collected from humans are scarce. Nonetheless, the national monitoring system and reporting is still absent in certain European countries. Romania has a high climatic and habitat heterogeneity favorable for the tick vectors and for developing vector-borne diseases and thus it is increasingly a focus of concern in our country. Therefore, the presence of previously unknown tick-borne pathogens in questing [10,11,12,13,14,15] and engorged ticks collected from the wild hosts have been reported [3,16,17,18,19,20,21,22,23]. The results of the present study highlight the common occurrence of tick-borne pathogens in *I. ricinus* collected from humans as well. Our data on prevalence is in line with reported statistics in certain European studies on ticks collected from humans [8,24,25,26]. In the same study period, Andersson et al. [8] reported a slightly lower average infection rate of certain tick-borne pathogens in *I. ricinus* collected from humans in another Romanian county (Sibiu); however, some species remained undetected (*B. lusitaniae*, *Babesia* spp.). Variations may possibly be explained by differences in sampling areas or even the sensitivity of the employed pathogen detection method.

The reported prevalence of *B. burgdorferi* s.l. infection in *I. ricinus* collected from humans in the present study (12.64%) is similar to the mean overall prevalence of 13.7% in Europe [27]. *B. afzelii*, mainly associated with rodents, and *B. garinii*, associated with birds, were also the most frequently detected spirochete species in previous Romanian studies [3,4,8,10,11,12,15]. The higher prevalence of *B. afzelii* might be related to the abundance of rodent-based enzootic cycles in the investigated area. It is known that the distribution and prevalence of *Borrelia* spp. in ticks show significant temporal and spatial variations. However, the infection prevalence of three spirochete species responsible for clinical diseases in humans, *B. afzelii*, *B. garinii* and *B. valaisiana*, was relatively in accordance with the pervious report elaborated by Briciu et al. [4]. In *I. ricinus* collected from humans in the same study area, a slightly higher infection rate was observed for *B. lusitaniae*, with an infection prevalence increasing in time and ranging between 0.19–2.49% (2010 vs. 2013–2015). The variations in prevalence of this spirochete species may be a result of climate change, since favorable conditions might increase the reservoir host density. *B. lusitaniae* is mainly associated with lizard hosts (*Lacerta agilis*, *L. viridis*), commonly found throughout our study areas [28].

Despite the sporadic geographic distribution of the relapsing fever group agent transmitted by *I. ricinus*, *B. miyamotoi* has been detected in several European countries since its first report in questing *I. ricinus* in Romania [13]. Regardless of the low number of case studies on *B. miyamotoi* infection, certain epidemiological surveys report co-infections with other spirochete species, probably due to the overlap of endemic areas for *B. miyamotoi* with species from *B. burgdorferi* s.l. complex. The existence of this spirochete was also confirmed in small mammals in our county [3] and in *I. ricinus* collected from humans in another Romanian county in the same study period [8]. Nonetheless, no human cases of *B. miyamotoi* infection have been reported so far in Romania.

Larvae of *I. ricinus* pose a potential infection risk and are able to transmit *B. burgdorferi* s.l. Moreover, a potentially higher contribution of larvae to the transmission of *B. miyamotoi* spirochetes to humans has been shown [29]. According to previous studies, larvae are responsible for 4–21.6% of human tick bites in Romania [4]. However, a significant number of larval ticks are not detected by people due to their size. In the present study the infection prevalence of *Borrelia* in larvae was 9.52%, shared equally by two species, *B. afzelii* and *B. miyamotoi*. The transovarial transmission of these spirochetes was demonstrated in previous studies [30,31], although it is generally considered to be low [30,31]. Despite this, there are multiple reports of *Borrelia* spp. in questing larvae of *I. ricinus* [12,30,32]. However, the detection of spirochetes DNA does not necessarily imply the viability of these bacteria.

Mixed infection with *Borrelia* spp./*N. mikurensis* seems to be common in *I. ricinus* [33]. *N. mikurensis* was the second most abundant bacterial species in our study. This emerging tick-borne pathogen transmitted by *I. ricinus* ticks poses a still unclear risk to public health. It is considered the responsible agent for neoehrlichiosis, a disease causing a systemic inflammatory infection in immunocompromised patients. Studies on the presence of *N. mikurensis* in *I. ricinus* ticks collected from humans across Europe reported prevalence between 0.5–8.1%. However, in Central Europe the infection incidence occurs in 6% of *I. ricinus* with an overall infection prevalence of 6.2% [8]. Human cases of infection with this pathogen have never been reported in our country; nonetheless, the first incidence of infection was reported in one *I. ricinus* tick collected from a patient [34], particularly, the *N. mikurensis*-positive tick was co-infected with *B. afzelii*. Recently, the same author [8] reported a slightly lower infection prevalence (1.7%) in ticks collected from humans in central part of Romania in the same study period.

Data on the prevalence of *Babesia* spp. in *I. ricinus* ticks collected from humans vary considerably. Babesiosis affects animals and humans and is transmitted by ticks. Human babesiosis in Europe is caused mainly by *B. divergens*, *B. venatorum* and occasionally by *B. microti* [35]. To date, no infection with *Babesia* spp. in *I. ricinus* collected from humans have been reported in Romania. While the reservoir role of *B. venatorum* is the roe deer, this species was also reported in questing *I. ricinus* ticks, wild ungulates and in ticks originating from dogs [36,37]. The pathogenic *B. microti*, reported in several European countries, is identified foremost in the present study in *I. ricinus* collected from humans. In Romania, other *Babesia* species have been reported in equines, dogs [36], *I. ricinus* collected from cattle [38], bats [33], roe deer and goats [39]. However, infection rate data are scarce and further studies are needed regarding the potential medical importance of *Babesia* spp. as a human pathogen in Romania.

*Bartonella* spp. was described to cause afebrile bacteraemia in humans and animals [40]. Certain animal-associated *Bartonella* spp. species were isolated from blood of patients bitten by ticks, *I. ricinus* [41,42,43,44,45], bats and their ticks [22,46,47]. Other bacterial pathogens causing tick-borne diseases, such as *F. tularensis* (causing tularemia) and *C. burnetii* (causing Q fever) have a wide spectrum of hosts including livestock, pets, birds, reptiles, wildlife and have also been detected in *I. ricinus* with relatively low prevalence. However, to our knowledge, only one study reports the presence of *F. tularensis* DNA in *I. ricinus* collected from roe deer and goats in Romania [39]. Epidemiological studies from certain European countries [48,49] show that the infection prevalence with these pathogens is relatively low. However, the role of *I. ricinus* in the transmission of the pathogens is debated [50,51]. The lack of *Bartonella* spp., *Francisella* spp. and *Coxiella* spp. in *I. ricinus* collected from humans in Romania in the present study may suggest that the infection risk with these tick-borne pathogens is very low but not negligible, which may be explained by the focal occurrence of these pathogens.

The present results revealed a variety of human pathogenic bacterial co-infections in ticks attached to humans. Molecular studies showed that multiple infections occur from 3.2% to 45% of *I. ricinus* ticks and have a major impact in bacterial fitness, and they may generate increased bacterial burden [52,53,54]. Frequently reported co-infections may result from feeding of ticks on one or multiple hosts carrying different pathogens. In the present study, *Borrelia* spp. were present in most of the co-infections. Meta-analysis studies focusing on the infection prevalence of tick-borne pathogens in *I. ricinus* ticks have shown that co-infections with the combination of *Borrelia* spp./*N. mikurensis* and *Borrelia* spp./*Babesia* spp. are relatively rare. In a small-scale study from 2019, *Borrelia* spp. and *N. mikurensis*, respectively *Borrelia* spp. and *Babesia* spp., occurred together in less than 1% of *I. ricinus* collected from Denmark [53]. The co-occurrence of different pathogens suggests that *I. ricinus* feeds on multiple hosts of diverse species during their life cycle.

Tick-borne pathogens showed different patterns over the years. Although the infection prevalence of the detected pathogens altered between the years in the present study, with the exclusion of *B. miyamotoi,* the presence of the species did not change with time. While *B. miyamotoi* reached the highest infection prevalence in the second study year, it was not detected over all the years. The DNA of this pathogen in the first year was not recorded. Significant annual differences were observed for *N. mikurensis* with a higher infection rate in the second study year. The recorded prevalence rates of *B. burgdorferi* s.l. and *Babesia* spp. had a continuous increase in time, without significant annual differences. A similar trend was observed for co-infections. The pathogen species diversity, the number of co-infections and pathogen associations have been increasing over the years. Certain parameters can potentially affect pathogen associations. Climate factors and social and environmental drivers significantly affect the distribution and prevalence of tick-borne diseases [55,56]. Changes in these factors influence the vector population, reservoir host spectrum density and tick-borne pathogen reproduction rates, which may contribute to an increased risk of human infection with pathogens.

## 4. Materials and Methods

### 4.1. Tick Collection and Species Identification

During 2013–2015, ticks from humans from Cluj-Napoca and surroundings were collected by personnel from a healthcare center (Infectious Diseases Clinic and Emergency Hospital).

A questionnaire regarding the time and exposure place was completed by each tick-bitten patient. Collected ticks were stored at −70 °C until further analysis and were referred to our laboratory for species identification based on morphological features using dichotomous keys [9] and molecular investigation of pathogens.

### 4.2. DNA Isolation

Genetic material isolation was performed individually from each *I. ricinus* tick using a commercial DNA extraction kit (Isolate II Genomic DNA kit; Bioline, London, UK) according to the manufacturer’s instructions. In order to identify possible cross-contamination, negative controls were used in each sample set during the procedure.

### 4.3. Tick-Borne Pathogen Detection

DNA samples were assessed for the presence of the following tick-borne pathogens: *Borrelia* spp., *N. mikurensis*, *Babesia* spp., *Bartonella* spp., *Coxiella* spp. and *Francisella* spp. Multiplex quantitative polymerase chain reaction (mqPCR) was used for evaluating the presence of *Borrelia* spp. in ticks. The gene encoding the outer surface protein A (*ospA*) was targeted for *B. burgdorferi* s.l. and a part of the flagellin B (*flab*) gene for *B. miyamotoi*. For *N. mikurensis* a simple quantitative PCR (qPCR) was performed. *Borrelia* spp. and *N. mikurensis* positive samples obtained by mqPCR and qPCR were amplified by convPCR using the primers and protocols described in Table 3. A nested-PCR (nPCR) amplifying the 18S rDNA of *Babesia* spp. was used in the screening. The detection of *Bartonella* spp., *C. burnetii* and *Francisella* spp. DNA was carried out by conventional-PCR (convPCR) assays. Targeted genes and protocols for each pathogen are described in Table 3. The qPCR reactions were performed in CFX96 Touch™ Real-Time PCR detection system (Bio-Rad, London, UK) using IQ Multiplex Powermix (Bio-Rad) in a final volume of 20 µL. The final volume of convPCR and nPCR was 25 µL; the reaction mixture contained 2x Red PCR master Mix (Rovalab GmBH, Teltow, Germany). The convPCR and nPCR were performed in T100™ Thermal Cycler (Bio-Rad). For quality control of PCR reactions, negative and positive controls were used.

### 4.4. DNA Sequencing

All the nPCR and convPCR-positive samples were sequenced at Macrogen Inc., Amsterdam, Netherlands. Nucleotide sequences were compared with those available in GenBank^TM^ using Basic Local Alignment Search Tool (BLAST) analysis and were submitted to GenBank under accession numbers MT145337-MT145340, MT345113, MT345115-MT345116, MT345158 and MT345316-MT345317.

### 4.5. Statistical Analysis

Epi Info^TM^ 2000 software (https://www.cdc.gov/epiinfo/) was used for statistical calculations. The infection prevalence of each pathogen and the 95% confidence interval were calculated and infection prevalence differentiated by developmental stages was analyzed using a chi-squared independence test.

## 5. Conclusions

The present survey serves as a status report for the north-western part of Romania and a three-year follow-up survey showing an increasing trend in the annual incidence of tick-borne pathogens in *I. ricinus*. We found that co-infections with different species of tick-associated pathogens are relatively common and therefore should be regarded in the future diagnosis of tick-borne diseases. Consequently, due to the lack of information on human infections with tick-borne pathogens in our country, the implementation of molecular diagnosis methods as a routine diagnosis assay is needed.

## Figures and Tables

**Table 1:** The prevalence of *Borrelia* spp., *N. mikurensis* and *Babesia* spp. in *I. ricinus* collected from humans.

Pathogen	Prevalence % (+/n; 95% CI)			
Year	Larvae	Nymphs	Female	Total
*B. afzelii*				
2013	-	5.80 (8/138; 2.54–11.10)	0 (0/16)	5.19 (8/154; 2.27–9.98)
2014	6.25 (1/16; 0.16–30.2)	2.54 (3/118; 0.53–7.25)	7.14 (3/42; 1.50–19.48)	3.98 (7/176; 1.61–8.02)
2015	0 (0/5)	6.83 (11/161; 3.46–11.90)	11.54 (3/26; 2.45–30.15)	7.29 (14/192; 4.04–11.93)
Average	4.76 (1/21; 0.12–23.82)	5.28 (22/417; 3.51–7.86)	7.14 (6/84; 2.67–14.90)	5.56 (29/522; 3.90–7.86)
*B. garinii*				
2013	-	3.62 (5/138; 1.19–8.25)	6.25 (1/16; 0.16–30.23)	3.9 (6/154; 1.44–8.29)
2014	0 (0/16)	5.08 (6/118; 1.89–10.74)	4.76 (2/42; 0.58–16.16)	4.55 (8/176; 1.98–8.76)
2015	0 (0/5)	0.62 (1/161; 0.02–3.41)	0 (0/26)	0.52 (1/192; 0.01–2.87)
Average	0 (0/21)	2.88 (12/417; 1.65–4.96)	3.57 (3/84; 0.74–10.08)	2.87 (15/522; 1.75–4.69)
*B. lusitaniae*				
2013	-	0 (0/138)	0 (0/16)	0 (0/154)
2014	0 (0/16)	5.93 (7/118; 2.42–11.84)	7.14 (3/42; 1.50–19.48)	5.68 (10/176; 2.76–10.20)
2015	0 (0/5)	1.86 (3/161; 0.39–5.35)	0 (0/26)	1.56 (3/192; 0.32–4.50)
Average	0 (0/21)	2.40 (10/417; 1.31–4.36)	3.57 (3/84; 0.74–10.08)	2.49 (13/522; 1.46–4.21)
*B. valaisiana*				
2013	-	0 (0/138)	0 (0/16)	0 (0/154)
2014	0 (0/16)	0 (0/161)	0 (0/42)	0 (0/16)
2015	0 (0/5)	4.35 (7/161; 1.77–8.75)	7.69 (2/26; 0.95–25.13)	4.69 (9/192; 2.17–8.71)
Average	0 (0/21)	1.68 (7/417; 0.82–3.42)	2.38 (2/84; 0.29–8.34)	1.72 (9/522; 0.91–3.24)
*B. miyamotoi*				
2013	-	0 (0/138)	0 (0/16)	0 (0/154)
2014	6.25 (1/16; 0.16–30.23)	2.54 (3/118; 0.53–7.25)	4.76 (2/42; 0.58–16.16)	3.41 (6/176; 1.26–7.27)
2015	0 (0/5)	1.24 (2/161; 0.15–4.42)	0 (0/26)	1.04 (2/192; 0.13–3.71)
Average	4.76 (1/21; 0.12–23.82)	1.20 (5/417; 0.51–2.78)	2.38 (2/84; 0.29–8.34)	1.53 (8/522; 0.78–2.99)
*N. mikurensis*				
2013	-	0.72 (1/138; 0.02–3.97)	0 (0/16)	0.65 (1/154; 0.02–3.56)
2014	0 (0/16)	9.32 (11/118; 4.75–16.07)	4.76 (2/42; 0.58–16.16)	7.39 (13/176; 3.99–12.30)
2015	0 (0/5)	9.94 (16/161; 5.79–15.64)	3.85 (1/26; 0.10–19.64)	8.85 (17/192; 5.24–13.80)
Average	0 (0/21)	6.71 (28/417; 4.69–9.53)	3.57 (3/84; 0.74–10.08)	5.94 (31/522; 4.21–8.31)
*B. microti*				
2013	-	0.72 (1/138; 0.02–3.97)	0 (0/16)	0.65 (1/154; 0.02–3.56)
2014	0 (0/16)	2.54 (3/118; 0.53–7.25)	0 (0/42)	1.70 (3/176; 0.35–4.90)
2015	0 (0/5)	3.73 (6/161; 1.38–7.93)	3.85 (1/26; 0.10–19.64)	3.65 (7/192; 1.48–7.37)
Average	0 (0/21)	2.40 (10/417; 1.31–4.36)	1.19 (1/84; 0.03–6.46)	2.11 (11/522; 1.18–3.73)
*B. venatorum*				
2013	-	0.72 (1/138; 0.02–3.97)	0 (0/16)	0.65 (1/154; 0.02–3.56)
2014	0 (0/16)	0.85 (1/118; 0.02–4.63)	0 (0/42)	0.57 (1/176; 0.01–3.12)
2015	0 (0/5)	1.24 (2/161; 0.15–4.42)	0 (0/26)	1.04 (2/192; 0.13–3.71)
Average	0 (0/21)	0.96 (4/417; 0.37–2.44)	0 (0/84)	0.77 (4/522; 0.30–195)
Total 2013	-	10.87 (15/138; 6.21–17.29)	6.25 (1/16; 0.16–30.23)	10.39 (16/154; 6.06–16.31)
Total 2014	12.5 (2/16; 1.55–38.35)	25.42 (30/118; 17.86–34.26)	28.57 (12/42; 15.72–44.58)	25.00 (44/176; 18.79–32.07)
Total 2015	0 (0/5)	26.71 (43/161; 20.05–32.24)	26.92 (7/26; 11.57–47.79)	26.04 (50/192; 19.99–32.85)
**TOTAL**	**9.52 (2/21; 1.17–30.38)**	**20.86 (88/417; 17.24–82.76)**	**23.82 (20/84; 15.19–34.35)**	**21.07 (110/522; 17.79–24.78)**

-: not collected; +/n: number of positive samples/total number of samples.

**Table 2:** Multiple infections with *Borrelia* spp., *N. mikurensis* and *Babesia* spp. in *I. ricinus* collected from humans in Romania.

Pathogens	Prevalence % (+/n; 95% CI)			
	2013	2014	2015	Total
*B. afzelii* + *B. microti*	6.25 (1/16; 0.16–30.23)	-	-	0.91 (1/110; 0.02–4.96)
*B. afzelii* + *N. mikurensis*	-	-	2 (1/50; 0.05–10.65)	0.91 (1/110; 0.02–4.96)
*B. garinii* + *N. mikurensis*	-	4.55 (2/44; 0.56–15.47	-	1.82 (2/110; 0.22–6.41)
*B. lusitaniae* + *N. mikurensis*	-	2.27 (1/44; 0.06–12.02)	-	0.91 (1/110; 0.02–4.96)
*B. valaisiana + N. mikurensis*	-	-	2 (1/50; 0.05–10.65)	0.91 (1/110; 0.02–4.96)
*B. valaisiana + N. mikurensis* + *B. venatorum*	-	-	2 (1/50; 0.05–10.65)	0.91 (1/110; 0.02–4.96)
*N. mikurensis* + *B. microti*	-	-	2 (1/50; 0.05–10.65)	0.91 (1/110; 0.02–4.96)
*N. mikurensis* + *B. venatorum*	-	2.27 (1/44; 0.06–12.02)	-	0.91 (1/110; 0.02–4.96)
**TOTAL**	**6.25 (1/16; 0.16–30.23)**	**9.09 (4/44; 2.53–21.67)**	**8 (4/50; 2.22–19.23)**	**8.18 (9/110; 3.81–14.96)**

-: not detected; +/n: number of co-infected samples/total number of positive samples with at least one pathogen.

**Table 3:** Targeted genes and primers used for the screening of tick-borne pathogens.

Pathogen	Primer name	Primer sequence (5′…′3)	Gene	PCR	References
*Borrelia* spp.	B_OspA_F	AATATTTATTGGGAATAGGTCTAA	*ospA*	mqPCR	[44]
	B_OspA_R	CTTTGTCTTTTTCTTTRCTTACA			
	B_OspA_P	FAM-AAGCAAAATGTTAGCAGCCTTGA-BHQ1			
	BMiya_F	AGAAGGTGCTCAAGCAG	*flaB*		[57]
	BMiya_R	TCGATCTTTGAAAGTGACATA T			
	BMiya_P	Cy5-AGCACAACAGGAGGGAGTTCAAGC-BHQ2			
	5SCB	GAGTTCGCGGGAGAGTAGGTTATTGCC	IGS	convPCR	[58]
	23SN	TCAGGGTACTTAGATGGTTCACTTCC			
	OspA F	GCAAAATGTTAGCAGCCTTGAT	*ospA*		
	OspA R	CTGTGTATTCAAGTCTGGTTCC			
	glpQ_BM_F	ATGGGTTCAAACA AAAAGTCACC	*glpQ*		[59]
	glpQ_BM_R	CCAGGGTCCAATTCCATCAGAATATTGTGCAAC			
*N. mikurensis*	NMikGroEL-F2a	CCTTGAAAATATAGCAAGATCAGGTAG	*groEL*	qPCR	[60]
	NMikGroEL-R2b	CCACCACGTAACTTATTTAGTACTAAAG			
	NMikGroEL-P2a	FAM-CCTCTACTAATTATTGCTGAAGATGTAGAAGGTGAAGC-BHQ1			
	NMik fo-groEL	GAAGYATAGTYTAGTATTTTTGTC		convPCR	
	NMik re-groEL	TTAACTTCTACTTCACTTGAACC			
*Babesia* spp.	BTH-1F	CCTGAGAAACGGCTACCACATCT	18S rRNA	nPCR	[37,61]
	BTH-1R	TTGCGACCATACTCCCCCCA			
	GF2	GTCTTGTAATTGGAATGATGG			
	GR2	CCAAAGACTTTGATTTCTCTC			
*Bartonella* spp.	443F	GCTATGTCTGCATTCTATCA	*glta*	nPCR	[62]
	1210R	GATCYTCAATCATTTCTTTCCA			
	CSH1F	GCGAATGAAGCGTGCCTAAA			
	BhCS.1137	AATGCAAAAAGAACAGTAAACA			
*F. tularensis*	TUL4-435	GCTGTATCATCATTTAATAAACTGCTG	17 kDa lipoprotein	convPCR	[63]
	TUL4-863	TTGGGAAGCTTGTATCATGGCACT			
*Coxiella* spp.	Trans B	CAAGAATGATCGTAACGATGCGC	IS1111		[64]
	Trans M	CTCGTAATCACCAATCGCTTCG			

mqPCR—multiplex-quantitative PCR, convPCR—conventional PCR, qPCR—quantitative PCR, nPCR nested PCR.

## Supplementary Materials

The following are available online at https://www.mdpi.com/2076-0817/9/5/390/s1, Table S1: Overall prevalence of tick-borne pathogens in *I. ricinus* ticks collected from humans, Table S2: Co-infections with different tick-borne pathogens in *I. ricinus* ticks collected from humans in Romania.
