# Supplementary material for: Multiple Tick-Borne Pathogens in Ixodes ricinus Ticks Collected from Humans in Romania
Source: Pathogens. 2020 May 19;9(5):390. doi: 10.3390/pathogens9050390 (PMC7281082; doi:10.3390/pathogens9050390)
Supplement: Supplementary file 1 [file pathogens-09-00390-s001.pdf]

Supplementary Material

Table S1. The overall prevalence of tick-borne pathogens in *I. ricinus* ticks collected from humans.

Pathogen	Prevalence % (+/n; 95% CI)			
	**Larvae**	**Nymphs**	**Female**	**Total**
A. phagocytophilum	4.76 (1/21; 0.12–23.82)	5.04 (21/417; 3.22–7.7)	8.33 (7/84; 3.42–16.42)	5.56 (29/522; 3.90–7.86)
B. microti	0 (0/21)	2.40 (10/417; 1.31–4.36)	1.19 (1/84; 0.03–6.46)	2.11 (11/522; 1.18–3.73)
B. venatorum	0 (0/21)	0.96 (4/417; 0.37–2.44)	0 (0/84)	0.77 (4/522; 0.30–1.95)
B. afzelii	4.76 (1/21; 0.12–23.82)	5.28 (22/417; 3.51–7.86)	7.14 (6/84; 2.67–14.90)	5.56 (29/522; 3.90–7.86)
B. garinii	0 (0/21)	2.88 (12/417; 1.65–4.96)	3.57 (3/84; 0.74–10.08)	2.87 (15/522; 1.75–4.69)
B. lusitaniae	0 (0/21)	2.40 (10/417; 1.31–4.36)	3.57 (3/84; 0.74–10.08)	2.49 (13/522; 1.46–4.21)
B. miyamotoi	4.76 (1/21; 0.12–23.82)	1.20 (5/417; 0.51–2.78)	2.38 (2/84; 0.29–8.34)	1.53 (8/522; 0.78–2.99)
B. valaisiana	0 (0/21)	1.68 (7/417; 0.82–3.42)	2.38 (2/84; 0.29–8.34)	1.72 (9/522; 0.91–3.24)
N. mikurensis	0 (0/21)	6.71 (28/417; 4.69–9.53)	3.57 (3/84; 0.74–10.08)	5.94 (31/522; 4.21–8.31)
R. helvetica	0 (0/21)	4.56 (19/417; 2.8–7.15)	7.14 (6/84; 2.67–14.9)	4.79 (25/522; 3.2–7.1)
R. monacensis	0 (0/21)	1.68 (7/417; 0.74–3.58)	1.19 (1/84; 0.03–6.46)	1.53 (8/522; 0.78–2.99)
**TOTAL**	**14.29 (3/21; 3.05–36.34)**	**30.46 (127/417; 26.23–35.03)**	**34.52 (29/84; 24.48–45.69)**	**30.46 (159/522; 26.67–34.54)**

-: not collected; +/n: number of positive samples/total number of samples.

Table S2. Co-infections with different tick-borne pathogens in *I. ricinus* ticks collected from humans in Romania.

Pathogens	Prevalence % (+/n; 95% CI)			
	**2013**	**2014**	**2015**	**Total**
A. phagocytophilum + *R. helvetica*	-	3.51 (2/57; 0.43–12.11)	1.54 (1/65; 0.04–8.28)	1.89 (3/159; 0.39–5.41)
A. phagocytophilum + *R. monacensis*	-	-	1.54 (1/65; 0.04–8.28)	0.63 (1/159; 0.02–3.45)
A. phagocytophilum + *B. afzelii*	-	1.75 (1/57; 0.04–9.39)	-	0.63 (1/159; 0.02–3.45)
A. phagocytophilum + *B. garinii*	2.7 (1/37; 0.07–14.16)			0.63 (1/159; 0.02–3.45)
A. phagocytophilum + *B. microti*	-	-	1.54 (1/65; 0.04–8.28)	0.63 (1/159; 0.02–3.45)
B. afzelii + *B. microti*	2.7 (1/37; 0.07–14.16)	-	-	0.63 (1/159; 0.02–3.45)
B. afzelii + *N. mikurensis*	-	-	1.54 (1/65; 0.04–8.28)	0.63 (1/159; 0.02–3.45)
B. afzelii + *R. helvetica*	-	-	1.54 (1/65; 0.04–8.28)	0.63 (1/159; 0.02–3.45)
B. garinii + *N. mikurensis*	-	3.51 (2/57; 0.43–12.11)	-	1.26 (2/159; 0.15–4.47)
B. garinii + *R. monacensis*	-	1.75 (1/57; 0.04–9.39)		0.63 (1/159; 0.02–3.45)
B. lusitaniae + *N. mikurensis*	-	1.75 (1/57; 0.04–9.39)	-	0.63 (1/159; 0.02–3.45)
B. valaisiana + *B. venatorum* + *N. mikurensis*	-	-	1.54 (1/65; 0.04–8.28)	0.63 (1/159; 0.02–3.45)
B. valaisiana + *N. mikurensis*	-	-	1.54 (1/65; 0.04–8.28)	0.63 (1/159; 0.02–3.45)
N. mikurensis + *B. microti*	-	-	1.54 (1/65; 0.04–8.28)	0.63 (1/159; 0.02–3.45)
N. mikurensis + *B. venatorum*	-	1.75 (1/57; 0.04–9.39)	-	0.63 (1/159; 0.02–3.45)
R. helvetica + *B. microti*	-	1.75 (1/57; 0.04–9.39)		0.63 (1/159; 0.02–3.45)
R. helvetica + *B. venatorum*	-	-	1.54 (1/65; 0.04–8.28)	0.63 (1/159; 0.02–3.45)
R. helvetica + *N. mikurensis*	-	-	4.62 (3/65; 0.96–12.9)	1.89 (3/159; 0.39–5.41)
**TOTAL**	**5.41 (2/37; 0.66–18.19)**	**15.79 (9/57; 7.48–27.87)**	**18.46 (12/65; 9.92–30.03)**	**14.47 (23/159; 9.40–20.91)**

-: not detected; +/n: number of co-infected samples/total number of positive samples with at least one pathogen.
